# Dacryoadenite tuberculeuse bilatérale: à propos d'un cas

**DOI:** 10.11604/pamj.2015.20.26.4663

**Published:** 2015-01-12

**Authors:** Bekkay Rezzoug, Nazih Tzili, Hassan Ali, Oubaida Elyamouni, Mahfoud El Khaoua, Hamza Elorch, Redouane El Hlimi, Abdallah El Hassan, Amina Berraho

**Affiliations:** 1 Service d'Ophtalmologie B, Hôpital des Spécialités, CHU Rabat, Maroc

**Keywords:** Dacryoadénite, tuberculose, glande lacrymale, inflammation, dacryoadenitis, tuberculosis, lacrimal gland, inflammation

## Abstract

La dacryoadénite tuberculeuse est une inflammation rare de la glande lacrymale causé par le bacille Mycobacterium tuberculosis. Elle pose un problème étiopathogénique et diagnostique. Nous rapportons dans cette observation le cas d'une dacryoadénite tuberculeuse bilatérale chez un jeune homme Marocain de 34 ans ayant présenté une tuberculose ganglionnaire et du cavum il y à 14 ans, ayant été confirmé par l'examen anatomopathologique. La tuberculose reste un diagnostic qui doit être toujours évoqué même dans les atteinte bilatérale surtout si il y a un antécédent personnel ou familiale positif de tuberculose. Le pronostic de cette affection est devenu favorable grâce au traitement antibacillaire précoce.

## Introduction

La tuberculose est une maladie infectieuse causée par le bacille Mycobacterium tuberculosis qui atteint le plus souvent les poumons, mais peut également affecter d´autres organes. De rares cas de dacryoadénite tuberculeuse ont été rapportés. Nous rapportons dans cette observation un cas de dacryoadénite tuberculeuse bilatérale.

## Patient et observation

**Figure 1:**
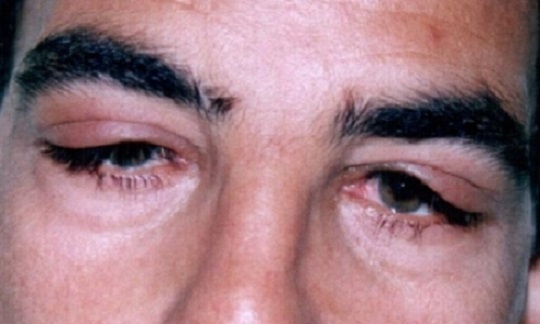
Le patient à son admission; tuméfaction des 2 angles supéro-externes de l'orbite droite et gauche sans signes inflammatoires associés

**Figure 2:**
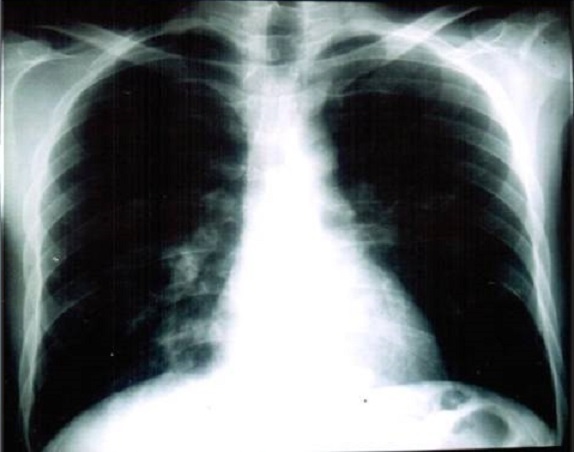
Radiographie thoracique montrant une image de caverne séquellaire parahilaire droite

**Figure 3:**
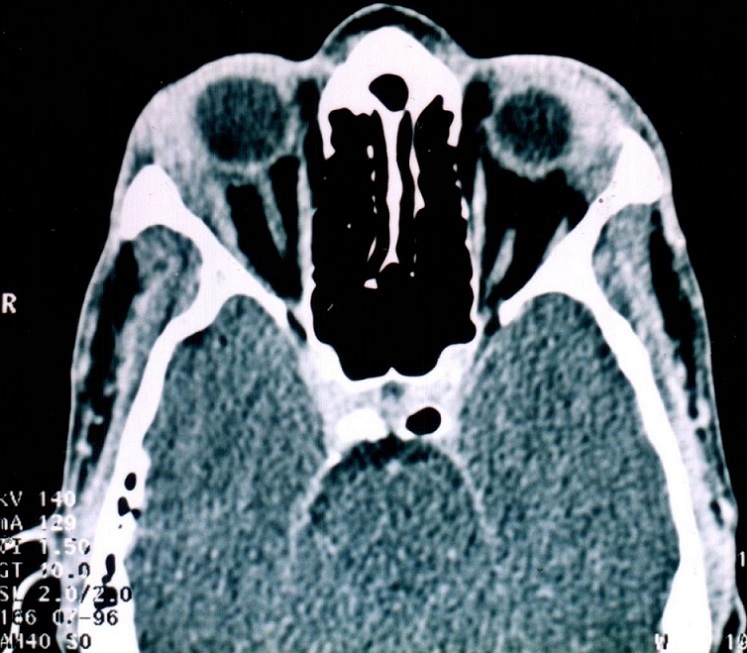
TDM orbito-cérébrale montrant une hypertrophie bilatérale, homogène, légèrement hyperdense des glandes lacrymales ne rehaussant pas au produit de contraste

**Figure 4:**
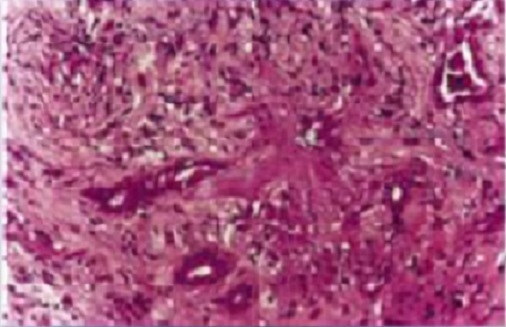
Image histologique du granulome épithéloide et gigantaux-cellulaire associé à une nécrose caséeuse

**Figure 5:**
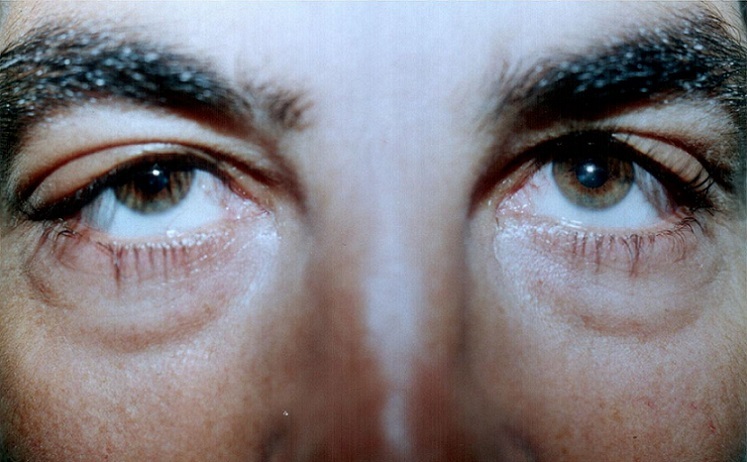
Le patient après traitement; nette amélioration

Nous rapportons le cas d'un jeune homme Marocain de 34 ans, traité il y'a 14 ans pour tuberculose ganglionnaire et du cavum, qui présente une tuméfaction des 2 angles supéro-externes de l'orbite droite et gauche sans signes inflammatoires associés (Figure 1). L'examen ophtalmologique trouve une exophtalmie bilatérale: de 25 mm au Hertel, non axile avec un globe dévié légèrement en bas et en dedans. L'espace oculo-orbitaire trouve une masse tumorale palpable au niveau l'angle supéro-externe, des deux cotés, à limites latérales imprécises et faisant corps avec la glande lacrymale. On note également une limitation des mouvements oculaires en haut et en dehors. Par ailleurs, Le globe oculaire est indemne avec une AV à 10/10 avec un fond ‘il normal des deux cotés. L'examen otorhinolaryngologique est sans particularité. Un bilan biologique a été demandé et montre: une vitesse de sédimentation (VS) élevée à 30 mm à la première heure, l'intradermoréaction à la tuberculine est positive à 22 mm, alors que la numération formule sanguine, le bilan phosphocalcique, le dosage du facteur rhumatoïde et la sérologie syphilitique sont normaux. La radiographie thoracique met en évidence une image de caverne tuberculeuse à droite (Figure 2). Une TDM orbito-cérébrale était demandée objectivant une hypertrophie bilatérale, homogène, légèrement hyperdense des glandes lacrymales ne rehaussant pas au produit de contraste (Figure 3). Une biopsie anatomopathologique de la tumeur confirme l'origine tuberculeuse en mettant en évidence un granulome épithéloide et gigantaux-cellulaire associé à une nécrose caséeuse (Figure 4). L’évolution sous traitement anti bacillaire de 6 mois et corticothérapie orale prolongée à 6 mois est marquée par la régression tumorale en 4 semaines (Figure 5) et l'absence de récidive après un recule de 4 ans.

## Discussion

Décrite pour la première fois par Abadie en 1881 [1], la dacryoadénite tuberculeuse est une affection rare même dans les pays endémique [2]. Elle touche surtout l'adulte jeune et l'atteinte bilatérale est exceptionnelle [3–5]. La dissémination est essentiellement hématogène [6] avec une localisation primaire pulmonaire comme c'est le cas chez notre patient, ganglionnaire ou osseuse, etc. La recherche d'un foyer primaire est alors systématique devant toute suspicion d'une dacryoadénite tuberculeuse. Dans notre contexte, le patient avait comme antécédent une tuberculose ganglionnaire et du cavum, ce qui nous a aidé à s'orienter vers une possibilité d'atteinte tuberculeuse de la glande lacrymale malgré sa rareté. Néanmoins, l'atteinte peut être primaire après contact avec des mains contaminées ou des crachats contenant des bacilles [7]. La dacryoadénite tuberculeuse se présente sous forme d'une dacryomégalie bilatérale et asymétrique entrainant une déformation de la paupière supérieure en forme de « S ». Sur le plan radiologique (scanner orbitaire), la dacryoadénite tuberculeuse peut être vu soit sous la forme d'un élargissement des glandes lacrymales ou des abcès [8]. Une destruction osseuse peut être mise en évidence [9]. Les autres causes de l´élargissement de la glande lacrymale comprennent le lymphome et la sarcoïdose, surtout dans l'atteinte bilatérale et ne montrent pas d´abcès orbitaire ou de destruction osseuse [8, 9]. L’étude anatomopathologique ou immuno-histochimique permet de poser le diagnostic étiologique surtout si l'atteinte est bilatérale sans contexte de contage tuberculeux. Le traitement des dacryoadénite tuberculeuse reste initialement médical, par des antibacillaires, avec un pronostic excellent [4, 10]

## Conclusion

La dacryoadénite tuberculeuse bilatérale est une entité clinique rare, elle touche l'adulte jeune mais aussi l'enfant. Son diagnostic bénéficie actuellement de la réalisation des biopsies et des progrès réalisés en matière d'immuno-histopathologie. Grace à la précocité du diagnostic, au traitement antibacillaire adapté et l'observance thérapeutique, le pronostic de cette affection est devenu favorable avec un risque faible de contamination communautaire.
